# Diluted Fecal Community Transplant Restores Clostridioides difficile Colonization Resistance to Antibiotic-Perturbed Murine Communities

**DOI:** 10.1128/mbio.01364-22

**Published:** 2022-08-01

**Authors:** Nicholas A. Lesniak, Sarah Tomkovich, Andrew Henry, Ana Taylor, Joanna Colovas, Lucas Bishop, Kathryn McBride, Patrick D. Schloss

**Affiliations:** a Department of Microbiology and Immunology, University of Michigan, Ann Arbor, Michigan, USA; University of Maryland, School of Medicine

**Keywords:** *Clostridioides difficile*, colonization resistance, microbial ecology, microbiome, fecal transplant, mice

## Abstract

Fecal communities transplanted into individuals can eliminate recurrent Clostridioides difficile infection (CDI) with high efficacy. However, this treatment is only used once CDI becomes resistant to antibiotics or has recurred multiple times. We sought to investigate whether a fecal community transplant (FCT) pretreatment could be used to prevent CDI altogether. We treated male C57BL/6 mice with either clindamycin, cefoperazone, or streptomycin and then inoculated them with the microbial community from untreated mice before challenge with C. difficile. We measured colonization and sequenced the V4 region of the 16S rRNA gene to understand the dynamics of the murine fecal community in response to the FCT and C. difficile challenge. Clindamycin-treated mice became colonized with C. difficile but cleared it naturally and did not benefit from the FCT. Cefoperazone-treated mice became colonized by C. difficile, but the FCT enabled clearance of C. difficile. In streptomycin-treated mice, the FCT was able to prevent C. difficile from colonizing. We then diluted the FCT and repeated the experiments. Cefoperazone-treated mice no longer cleared C. difficile. However, streptomycin-treated mice colonized with 1:10^2^ dilutions resisted C. difficile colonization. Streptomycin-treated mice that received an FCT diluted 1:10^3^ became colonized with C. difficile but later cleared the infection. In streptomycin-treated mice, inhibition of C. difficile was associated with increased relative abundance of a group of bacteria related to *Porphyromonadaceae* and *Lachnospiraceae*. These data demonstrate that C. difficile colonization resistance can be restored to a susceptible community with an FCT as long as it complements the missing populations.

## INTRODUCTION

The process by which gut bacteria prevent Clostridioides difficile and other pathogens from infecting and persisting in the intestine is known as colonization resistance (1). Antibiotic-induced disruption of the gut bacterial community breaks down colonization resistance and is a major risk factor for C. difficile infection (CDI) (2). Gut bacteria inhibit C. difficile through the production of bacteriocins, modulation of available bile acids, competition for nutrients, production of short-chain fatty acids, and alteration of the integrity of the mucus layer (1). After the initial CDI is cleared via antibiotics, patients can become reinfected. When CDI recurs more than once, the gut bacterial community from a healthy person typically is used to restore the gut community in the patient with recurrent CDI (3). Fecal microbiota transplant (FMT) is effective, but 10 to 20% of people that receive an FMT will still have another CDI (4). Additionally, transfer of a whole fecal community can incidentally transfer pathogens and cause adverse outcomes (5). While FMT is effective at curing recurrent CDI, it also has risks that must be considered.

The benefits and risks of FMT have led to the development of reduced bacterial communities to treat CDI. Synthetic communities are more defined than an FMT, making them easier to regulate as a drug. Tvede and Rask-Madsen were the first to successfully treat CDI with a community of isolates cultured from human feces (6). More recently, Lawley et al. analyzed murine experiments and the fecal communities from patients with CDI to develop a synthetic community of six isolates to inhibit C. difficile colonization (7). Reduced communities derived from human fecal communities by methods such as selective isolation of spores or culturing bacteria have cured recurrent CDI in their initial application (8–10). Although a recent phase 2 trial of SER-109, a spore-based treatment, failed its phase 2 clinical trial (11), these therapies have the potential to offer the benefits of FMT without the associated risks but are only used once a patient has had multiple CDIs. For these to be successful, we need a better approach to identify candidate bacterial populations. Recently, an autologous FMT was shown to be effective at restoring the gut microbiota in allogeneic hematopoietic stem cell transplantation patients and prevented future complications, such as systemic infections (12). It is unclear whether a treatment similar to an autologous FMT or reduced bacterial communities could be used to restore susceptible communities and prevent CDI (13).

Because FMT is often sufficient to restore colonization resistance to people with a current infection, we hypothesized that a fecal community should be sufficient to restore colonization resistance to an uninfected community. Therefore, we tested whether a fecal community transplant (FCT) pretreatment would prevent or clear C. difficile colonization and how variation in susceptibility to C. difficile infection would affect the effectiveness of FCT pretreatment. After testing the same FCT pretreatment across different antibiotic-induced susceptibilities, we sought to determine whether diluted FCT pretreatment could maintain the inhibition of C. difficile colonization and identify the bacterial populations associated with colonization resistance and clearance.

## RESULTS

### The effect of fecal transplant on C. difficile colonization was not consistent across antibiotic treatments.

**FIG 1:**
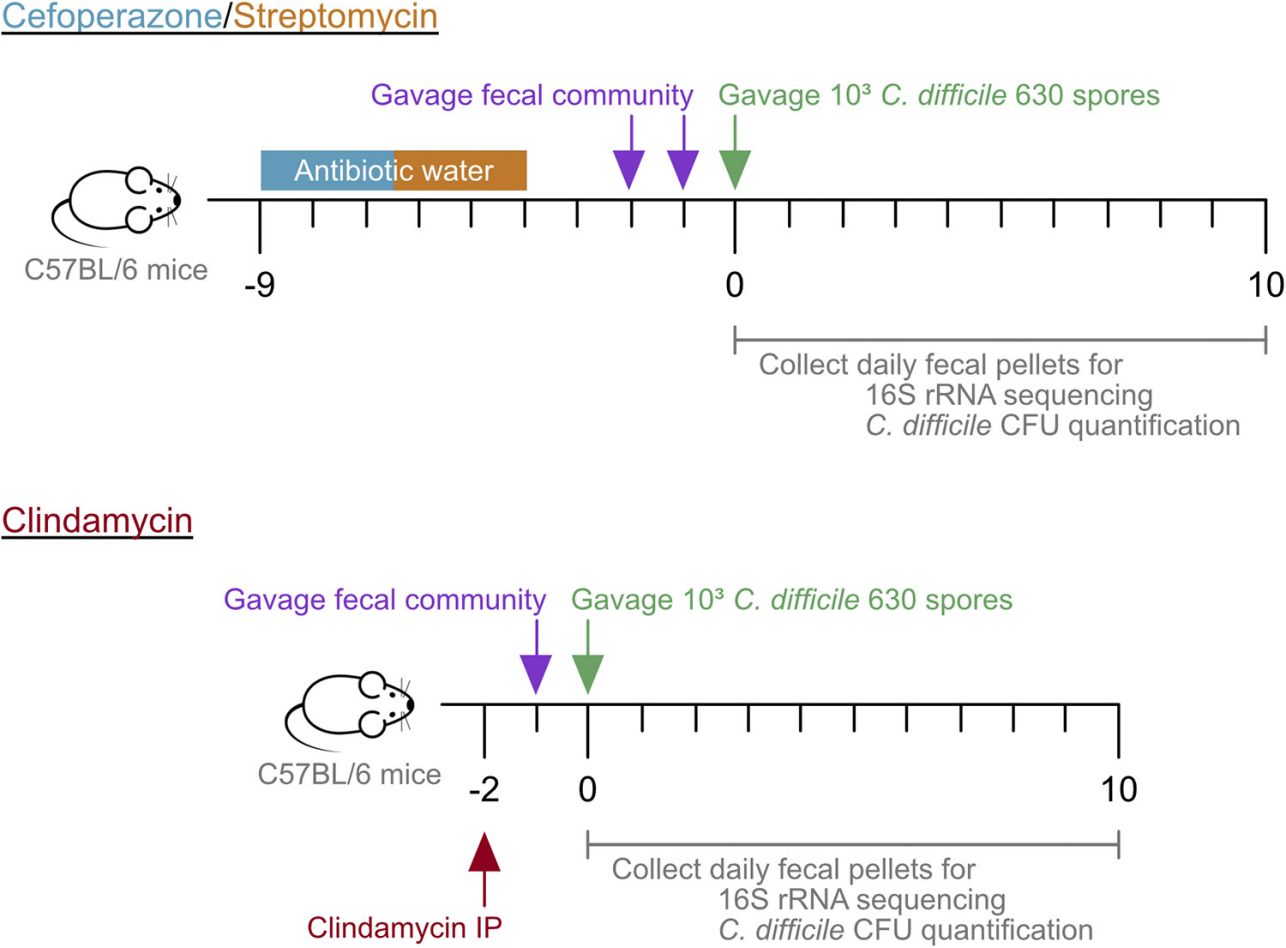
Mouse experiment timeline. Mice were given water with cefoperazone (0.5 mg/mL) or streptomycin (5 mg/mL) for 5 days. The mice were then given untreated water for the remainder of the experiment. Two days after the antibiotic water was removed, mice were given by oral gavage 100 μL of PBS or fecal community once a day for 2 days. The following day, the mice were challenged with 10^3^ C. difficile 630 spores. Alternatively, mice were given an intraperitoneal (IP) injection of clindamycin (10 mg/kg) 2 days prior to C. difficile infection. At 24 h later, mice were given by oral gavage 100 μL of PBS or fecal community. The following day, the mice were challenged with 10^3^ C. difficile 630 spores. Fecal pellets were collected prior to treatment (day −9 for cefoperazone/streptomycin, day −2 for clindamycin), cessation of antibiotics (day −2 for cefoperazone/streptomycin, day −1 for clindamycin), prior to C. difficile infection (day 0), and each of the following 10 days.

**FIG 2:**
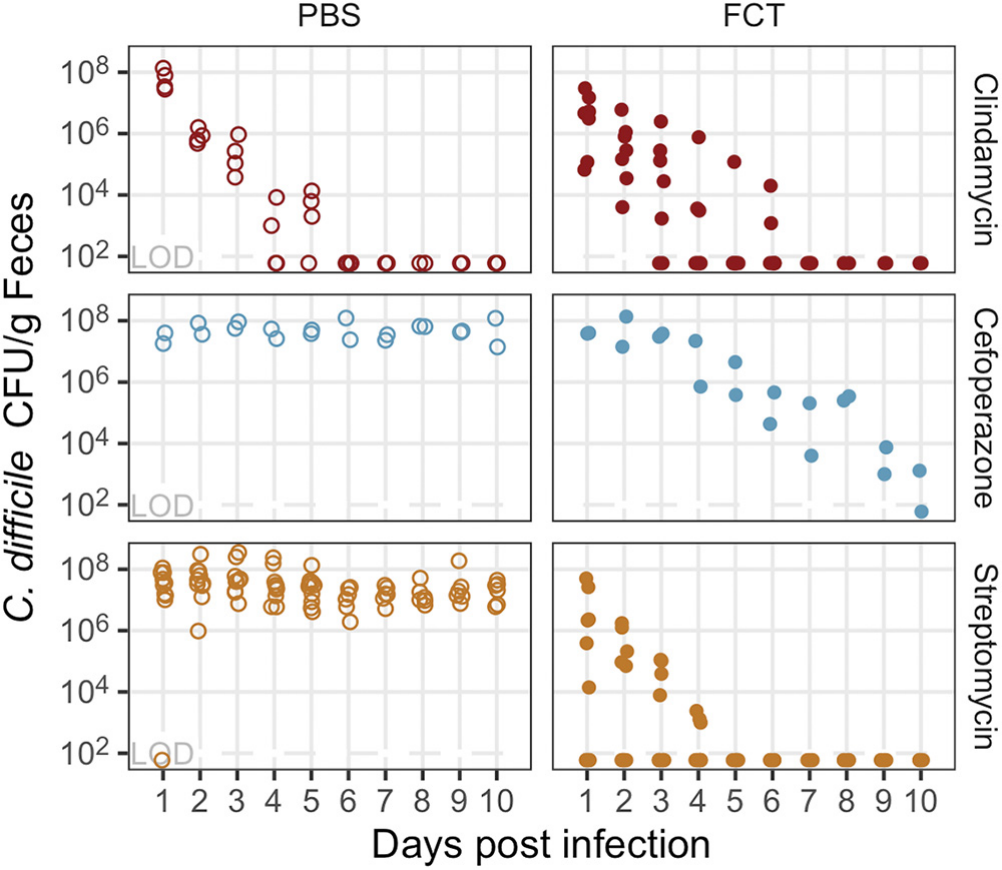
Fecal community transplant inhibited C. difficile colonization in mice treated with cefoperazone or streptomycin. The numbers of C. difficile CFU/g of feces for mice treated with clindamycin (red points), cefoperazone (blue points), or streptomycin (orange points) are shown. Mice were given by oral gavage either PBS (open circles) or fecal community transplant (FCT) (filled circles) prior to the C. difficile infection. Each point represents an individual mouse (clindamycin-PBS, *n* = 4, or clindamycin-FCT, *n* = 7; cefoperazone-PBS, *n* = 2, or cefoperazone-FCT, *n* = 2; streptomycin-PBS, *n* = 10, or streptomycin-FCT, *n* = 14). LOD, limit of detection.

Our previous research demonstrated that when mice were perturbed with different antibiotics, there were antibiotic-specific changes to the microbial community that resulted in different levels of colonization and clearance of Clostridioides difficile infection (14). Because each of these treatments opened different niche spaces that C. difficile could fill, we hypothesized that the resulting community varied in the types of bacteria required to recover colonization resistance. To test the ability of the murine communities to recover colonization resistance, we treated conventionally raised specific-pathogen-free (SPF) C57BL/6 mice with either clindamycin, cefoperazone, or streptomycin. After a short recovery period, the mice were given either phosphate-saline buffer (PBS) or a fecal community transplant via oral gavage (Fig. 1). The fecal community was obtained from untreated mice. One day after receiving the FCT, the mice were challenged with 10^3^ C. difficile 630 spores. One day after the challenge, mice that were treated with either clindamycin or cefoperazone and that received the FCT pretreatment had similar numbers of C. difficile CFU as those that received PBS. Among the clindamycin-treated mice, C. difficile colonization was cleared at similar rates regardless of whether the mice received the FCT or PBS pretreatment (Fig. 2). For cefoperazone-treated mice, C. difficile colonized all of the mice, but the mice that received the FCT pretreatment cleared the infection (Fig. 2). For the streptomycin-treated mice, the FCT pretreatment resulted in either no detectable C. difficile colonization (8 of 14) or an infection that the community cleared within 5 days (Fig. 2). For mice that normally would have had a persistent infection, the FCT enabled them to clear the infection, and in the streptomycin-treated mice, it was able to prevent infection entirely for some mice.

### Diluted fecal communities prevented colonization and promoted clearance for streptomycin-treated mice.

**FIG 3:**
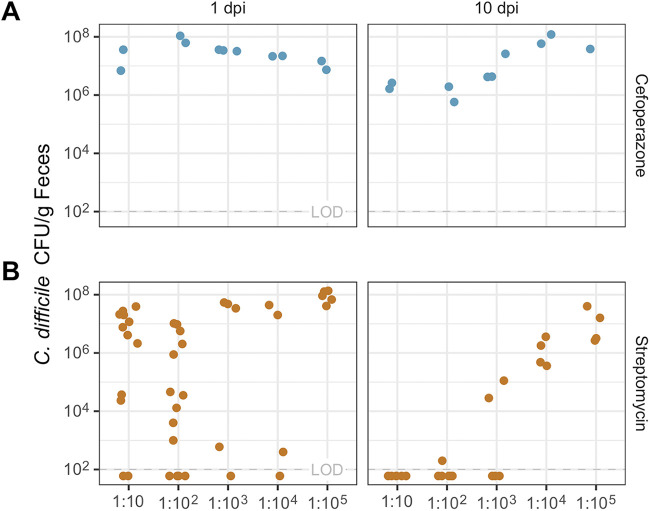
Diluted FCT inhibited C. difficile colonization in mice treated with streptomycin. The numbers of C. difficile CFU/g of feces for mice treated with cefoperazone (blue points) (A) or streptomycin (orange points) (B) are shown. Mice were given by oral gavage a dilution of FCT (1:10 to 1:10^5^) prior to C. difficile infection, and then the numbers of CFU/g of feces were counted at 1 day postinfection (dpi) and 10 dpi. Each point represents an individual mouse (for cefoperazone at a 1:10 dilution, *n* = 2; at 1:10^2^, *n* = 2; at 1:10^3^, *n* = 3; at 1:10^4^, *n* = 2; and at 1:10^5^, *n* = 2; for streptomycin at a 1:10 dilution, *n* = 12; at 1:10^2^, *n* = 14; 1:10^3^, *n* = 5; 1:10^4^, *n* = 4; and 1:10^5^, *n* = 5). LOD, limit of detection.

Next, we sought to test whether mice that received a diluted FCT pretreatment could still benefit. To identify the minimally effective dilution, we repeated the same experimental design (Fig. 1) with the FCT diluted serially down to 1:10^5^. Since the FCT pretreatment had no detected effect in clindamycin-treated mice, we did not study those mice further. Cefoperazone-treated mice pretreated with diluted FCT, at dilutions of 1:10 and lower, were not affected and were colonized throughout the experiment (Fig. 3). Streptomycin-treated mice pretreated with diluted FCT either regained colonization resistance or were enabled to clear C. difficile. The streptomycin-treated mice pretreated with FCT as dilute as 1:10^3^ cleared C. difficile. Some streptomycin-treated mice pretreated with FCT as dilute as 1:10^2^ had no C. difficile CFU detected throughout the length of the experiment. While more mice pretreated with the lower FCT dilutions were colonized (with undiluted FCT, 6 of 14 were colonized; with a 1:10 dilution, 10 of 12 were colonized; and with a 1:10^2^ dilution, 10 of 14 were colonized), the colonized mice that received the lower dilutions were still able to clear C. difficile (see Fig. S1 in the supplemental material). Thus, the reduced fecal communities from the diluted FCT were able to restore colonization resistance and promote clearance of C. difficile in streptomycin-treated mice.

10.1128/mbio.01364-22.1 FIG S1 C. difficile colonization dynamics in streptomycin-treated mice across all prophylactic transplant treatments. The numbers of C. difficile CFU/g of feces from streptomycin-treated mice given by oral gavage either PBS, fecal community transplant (FCT), or diluted FCT (1:10 to 1:10^5^) prior to the C. difficile infection are shown. Each semitransparent line represents an individual mouse. Mice were challenged with 10^3^ C. difficile 630 spores on day 0. Lines are grouped by the transplant treatment received. LOD, limit of detection. Download FIG S1, TIF file, 0.5 MB. Copyright © 2022 Lesniak et al. 2022 Lesniak et al. https://creativecommons.org/licenses/by/4.0/ This content is distributed under the terms of the Creative Commons Attribution 4.0 International license.

The reduced fecal communities of the diluted FCT may have reduced abundance and membership. We compared the FCT communities to determine the differences between the dilutions. The most significant difference between the communities of the FCT and its dilutions was the quantity of the 16S rRNA gene, which decreased monotonically (Fig. S2D). The FCT dilutions of 1:10^3^ to 1:10^5^ had few samples with sufficient sequencing depth to provide bacterial community information. The FCT and its dilutions were not significantly different in either α-diversity (number of operational taxonomic units [OTUs] [*S*_obs_] or inverse Simpson diversity index) or β-diversity (θ_YC_) (Fig. S2A to C). Populations of *Acetatifactor*, *Enterobacteriaceae*, *Lactobacillus*, *Ruminococcaceae*, and *Turicibacter* correlated with the FCT dilution factor (Fig. S2E). Overall, the abundance of the bacteria appeared to be the largest difference between FCT and its dilutions.

10.1128/mbio.01364-22.2 FIG S2 Diversity and quantification of fecal community dilutions used for prophylactic transplants in antibiotic-treated mice. (A to C) Diversity of fecal community dilutions. α-diversity was measured by *S*_obs_ (A) and the inverse Simpson diversity index (B) for undiluted fecal community (FCT) and diluted fecal communities (1:10 to 1:10^5^). Points represent individual samples. (C) β-diversity, measured by θ_YC_, of community structure of feces collected from untreated mice, undiluted fecal community (FCT), and diluted fecal communities (1:10 to 1:10^5^) compared to that of untreated feces. Points represent median values, and lines represent the interquartile range. (D) Quantification cycle (*C_q_*) values for qPCR of FCT and its dilutions for eubacterial 16S rRNA gene. Points represent median values, and lines represent the interquartile range. (E) Relative abundances of bacterial taxonomic groups that significantly correlate with fecal community dilutions (undiluted FCT to 1:10^3^) by Spearman’s correlation. Points represent individual samples. An asterisk indicates that the bacterial taxonomic group was unclassified at a lower classification rank. Download FIG S2, TIF file, 0.5 MB. Copyright © 2022 Lesniak et al. 2022 Lesniak et al. https://creativecommons.org/licenses/by/4.0/ This content is distributed under the terms of the Creative Commons Attribution 4.0 International license.

### Murine gut bacterial communities had not recovered their diversity by the time of C. difficile challenge.

**FIG 4:**
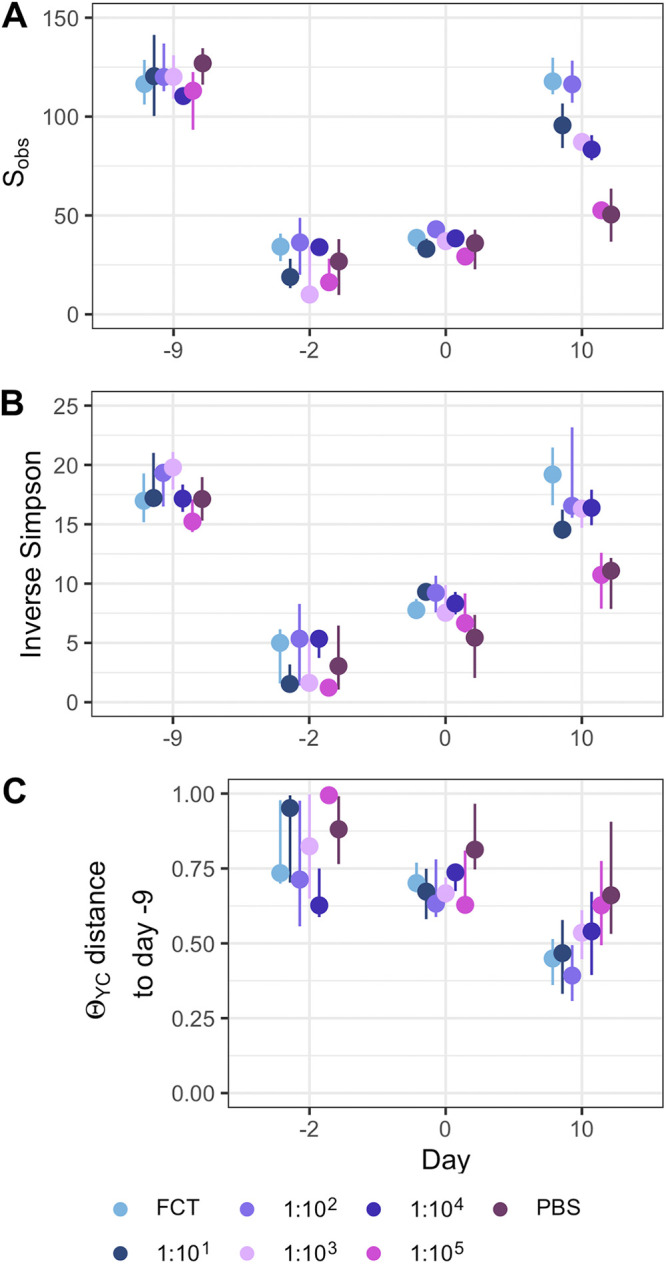
The diversity of the murine gut bacterial community had not recovered at the time of C. difficile infection in streptomycin-treated mice. α-diversity, measured by *S*_obs_ (A) and the inverse Simpson diversity index (B), prior to the start of antibiotic treatment (day −9), before fecal community transplant (day −2), after fecal community transplant on the day of C. difficile challenge (day 0), and at the end of the experiment (day 10). (C) β-diversity, measured by θ_YC_, indicates the distance between community structures on day -2, 0, or 10 and the community prior to antibiotic treatment (day −9) of that individual. Data are grouped by the transplant received, undiluted fecal community (FCT), diluted fecal community (1:10^1^ to 1:10^5^), or PBS. Points represent median values, and lines represent the interquartile range.

To elucidate the effects of the fecal community dilution on the murine gut bacterial community and C. difficile infection, we sequenced the V4 region of the 16S rRNA gene from the fecal community. For the gut communities, in comparison to the initial community (day −9), FCT pretreatment did not result in a significant recovery of diversity at the time of C. difficile challenge (day 0) for cefoperazone-treated mice (Fig. S3) or streptomycin-treated mice (Fig. 4). At the end of the experiment (day 10), the gut bacterial communities were more similar to their initial community in α-diversity (number of OTUs [*S*_obs_] and inverse Simpson diversity index) and β-diversity (θ_YC_). The mice pretreated with less-dilute FCTs had community structures most similar to the initial one, whereas the mice pretreated with more-dilute FCTs showed little recovery of diversity, similar to the mice given PBS. Thus, the less-dilute FCT treatments did not result in restoration of preantibiotic treatment community diversity at the time of C. difficile challenge but were sufficient to affect C. difficile colonization. This would suggest that the effect was driven by the most abundant populations.

10.1128/mbio.01364-22.3 FIG S3 Diversity of the murine gut bacterial community was not recovered at the time of C. difficile infection in cefoperazone-treated mice. Diversity changes through experiments with cefoperazone-treated mice. (A and B) α-diversity was measured by *S*_obs_ (A) and the inverse Simpson diversity index (B) prior to beginning antibiotic treatment (day −9), before fecal community transplant (day −2), after fecal community transplant on the day of C. difficile infection (day 0), and at the end of the experiment (day 10). (C) β-diversity, measured by θ_YC_, of the distance between community structures on day −2, 0, or 10 and the community prior to antibiotic treatment (day −9) of that individual. Data are grouped by the transplant received, undiluted fecal community (FCT), diluted fecal community (1:10^1^ to 1:10^5^), or PBS. Points represent individual mice. Download FIG S3, TIF file, 0.2 MB. Copyright © 2022 Lesniak et al. 2022 Lesniak et al. https://creativecommons.org/licenses/by/4.0/ This content is distributed under the terms of the Creative Commons Attribution 4.0 International license.

### Gut bacterial community members are differentially abundant in streptomycin-treated mice resistant to colonization.

**FIG 5:**
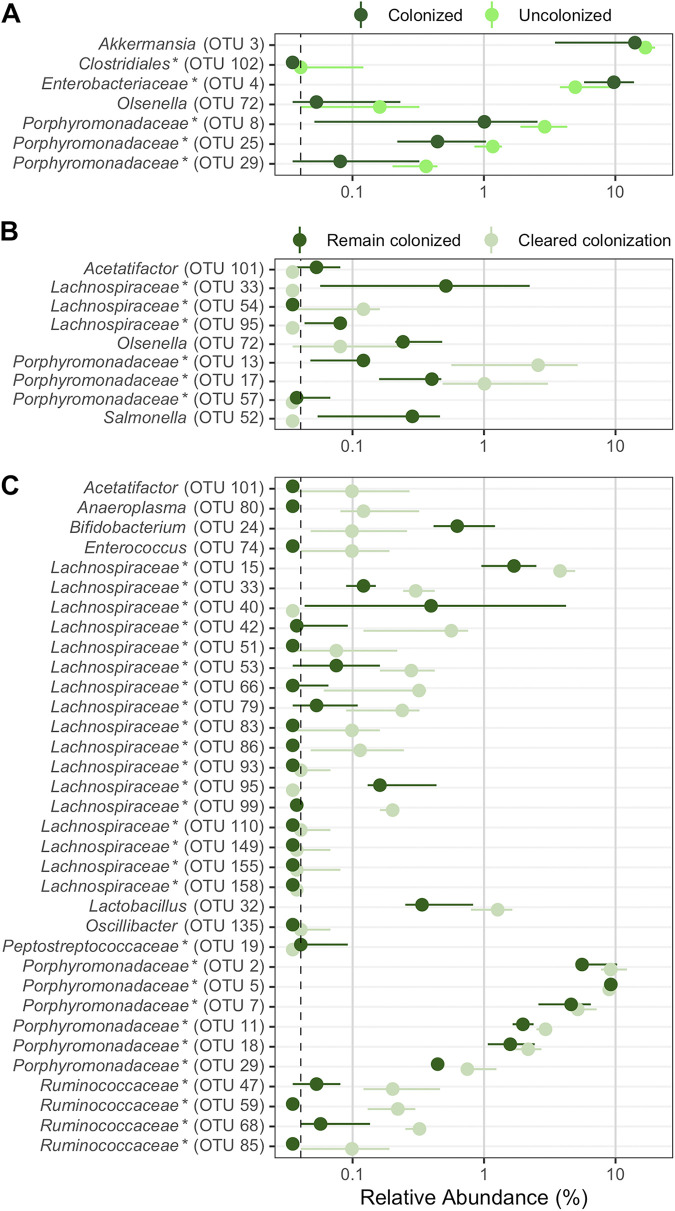
Bacterial community OTUs differentially abundant in streptomycin-treated mice that resisted or cleared colonization. Murine gut bacterial community OTUs that were significantly different by LEfSe analysis are shown. (A and B) OTUs from streptomycin-treated mice at the time of C. difficile challenge (day 0) which were differentially abundant between mice that were colonized (dark green) and those that were not (no detectable CFU throughout the experiment; bright green) (A) or mice that remained colonized (dark green) and those that cleared colonization (CFU reduced to below the limit of detection by the end of the experiment; light green) (B). (C) OTUs from streptomycin-treated mice at the end of the experiment (day 10) that were differentially abundant between mice that remained colonized (dark green) and those that cleared colonization (CFU reduced to below the limit of detection by the end of the experiment; faint green). Points represent median values, and lines represent the interquartile range. A dashed vertical line indicates the limit of detection. OTUs are ordered alphabetically. An asterisk indicates that the OTU was unclassified at a lower classification rank.

Although there were no significant differences in diversity at the time of challenge, we next investigated how the individual bacterial populations were different in the uncolonized streptomycin-treated mice pretreated with FCT. We used linear discriminant analysis (LDA) effect size (LEfSe) analysis to identify OTUs within the fecal bacterial communities from the streptomycin-treated mice that were differentially abundant between uncolonized and colonized mice. The antibiotic treatment significantly altered 99 OTUs (Fig. S5), but on the day of C. difficile challenge, only 7 OTUs were differentially abundant between colonized and uncolonized communities (Fig. 5A). Communities resistant to C. difficile colonization had more-abundant populations of OTUs related to *Akkermansia*, *Clostridiales*, *Olsenella*, and *Porphyromonadaceae* and less-abundant populations of an OTU related to *Enterobacteriaceae*. Thus, a small portion of OTUs, relative to the changes due to streptomycin treatment, were differentially abundant in mice that resisted C. difficile colonization compared to those that were colonized.

10.1128/mbio.01364-22.5 FIG S5 Murine gut bacterial community OTUs differentially abundant with streptomycin treatment. Murine gut bacterial community OTUs that were significantly different by LEfSe analysis between untreated mice (initial; black) and mice after 5 days of water with streptomycin (5 mg/mL) and 2 days of untreated water (after streptomycin; orange). Large bold points represent the group median. Small, semitransparent points represent an individual mouse. A gray arrow indicates the direction the relative abundance shifted with the streptomycin treatment. The left plot displays OTUs with a median relative abundance greater than 0.1%, and the right plot displays OTUs with a median relative abundance lower than 0.1%. A dashed vertical line indicates the limit of detection. OTUs are ordered alphabetically. An asterisk indicates that the OTU was unclassified at a lower classification rank. Download FIG S5, TIF file, 2.5 MB. Copyright © 2022 Lesniak et al. 2022 Lesniak et al. https://creativecommons.org/licenses/by/4.0/ This content is distributed under the terms of the Creative Commons Attribution 4.0 International license.

### Murine gut bacterial communities that cleared C. difficile colonization were more similar to the initial community.

To better understand the differences in streptomycin-treated murine fecal community that contributed to C. difficile clearance, we compared the communities that cleared C. difficile to those that did not at the time of challenge and 10 days postinfection. Communities from mice that cleared colonization were more similar to their initial community at the end of the experiment than the mice that remained colonized (Fig. S4). At the time of C. difficile challenge, 9 OTUs were differentially abundant between communities that remained colonized and those that cleared colonization (Fig. 5B). Communities that cleared C. difficile colonization had more-abundant populations of OTUs related to *Porphyromonadaceae* and *Lachnospiraceae* and less-abundant populations of OTUs related to *Acetatifactor*, *Lachnospiraceae*, *Olsenella*, *Porphyromonadaceae*, and Salmonella. At the end of the experiment, 29 of the 34 differentially abundant OTUs were more abundant in the mice that were able to clear the colonization (Fig. 5C). The relative abundance of OTUs related to *Acetatifactor*, *Anaeroplasma*, *Enterococcus*, *Lachnospiraceae*, *Lactobacillus*, *Porphyromonadaceae*, and *Ruminococcaceae* was higher in communities that cleared the colonization, recovering in abundance from the streptomycin treatment. Multiple OTUs related to *Lachnospiraceae* and *Porphyromonadaceae* (*n* = 14 and *n* = 5, respectively) were significant and accounted for greater portions of the community (more than 10%). However, one *Porphyromonadaceae* population (OTU 5) and two *Lachnospiraceae*-related populations (OTUs 40 and 95) were more abundant in the mice that remain colonized. Thus, as more of the gut bacterial members returned to their initial abundance, there was a greater likelihood of clearing C. difficile.

10.1128/mbio.01364-22.4 FIG S4 The gut bacterial community of streptomycin-treated mice that cleared colonization was more similar to their initial community. Diversity differences by outcome in streptomycin-treated mice. β-diversity, measured by θ_YC_, of the distance between community structures on day −2, 0, or 10 and the community prior to antibiotic treatment (day −9) of that individual. Data are grouped by the outcome, i.e., cleared colonization (faint green) or remain colonized (dark green). Points represent median values, and lines represent the interquartile range. An asterisk indicates significant difference by the Wilcoxon rank sum test with Bonferroni’s correction. Download FIG S4, TIF file, 0.1 MB. Copyright © 2022 Lesniak et al. 2022 Lesniak et al. https://creativecommons.org/licenses/by/4.0/ This content is distributed under the terms of the Creative Commons Attribution 4.0 International license.

### Negative associations dominated the interactions between the gut bacterial community and C. difficile in streptomycin-treated mice.

**FIG 6:**
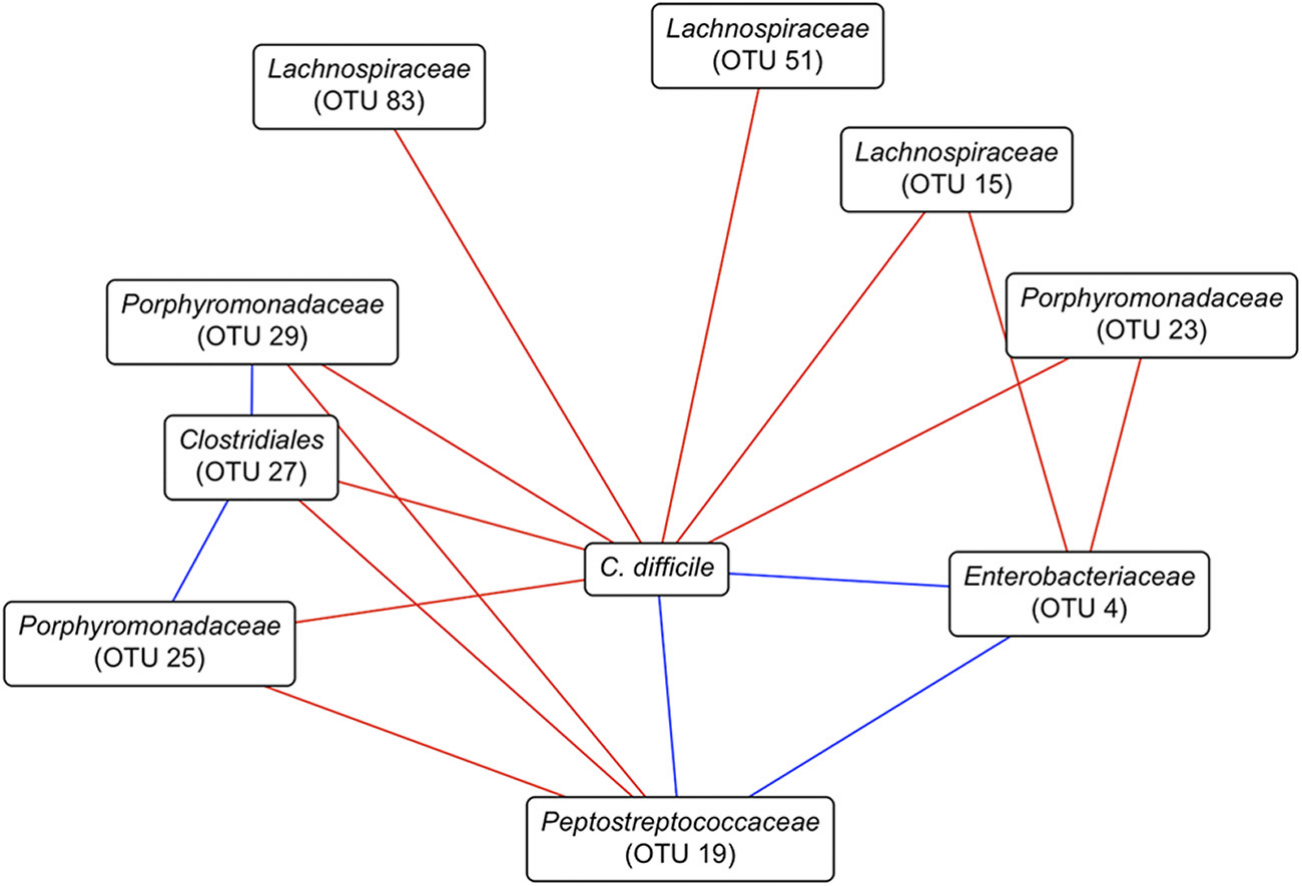
Streptomycin-treated murine fecal community associations with C. difficile. The network was constructed with SpiecEasi from the OTU relative abundances and C. difficile CFU data from days 1 through 5 after C. difficile infection. Red lines indicate negative associations, and blue lines indicate positive associations. C. difficile is based on CFU counts, and *Peptostreptococcaceae* (OTU 19), the OTU most closely related to C. difficile, is based on sequence counts. Only the C. difficile subnetwork is shown.

In streptomycin-treated mice, pretreatment with FCT and its dilutions had different effects on the bacterial community members, which resulted in different community relative abundances and C. difficile colonization dynamics. We quantified the relationships occurring throughout this experiment by using SPIEC-EASI (sparse inverse covariance estimation for ecological association inference) to construct a conditional independence network. Here, we focused on the associations of the C. difficile subnetwork (Fig. 6). C. difficile CFU had positive associations with populations of OTUs related to *Enterobacteriaceae* (OTU 4) and *Peptostreptococcaceae* (OTU 19). OTUs related to *Clostridiales* (OTU 27), *Lachnospiraceae* (OTUs 15, 51, and 83), and *Porphyromonadaceae* (OTUs 23, 25, and 29) had negative associations with C. difficile, as well as the OTUs related to *Enterobacteriaceae* and *Peptostreptococcaceae*. Overall, the majority of the associations between C. difficile and the gut bacterial community in streptomycin-treated mice were negative. This suggests that this subset of the community may drive the inhibition of C. difficile in streptomycin-treated communities.

## DISCUSSION

Transplanting the fecal community from untreated mice to antibiotic-treated mice prior to challenge with C. difficile varied in effectiveness based on the antibiotic treatment. This indicated that FCT pretreatment can prevent C. difficile colonization in an antibiotic-specific manner. Additionally, by diluting the FCT, we were able to narrow the community changes responsible for the effect to the most abundant OTUs. Overall, these results show that a reduced fecal community can assist a perturbed microbiota in preventing or resisting C. difficile colonization but that the effect was dependent on the antibiotic that was given.

By diluting the FCT, we were able to narrow the definition of the minimal community features that restored colonization resistance. Bacterial interactions with C. difficile were associated with the identity, abundance, and functions of adjacent bacteria. Ghimire et al. recently showed individual species that inhibited C. difficile in coculture, but when other inhibitory species were added, the overall effect on C. difficile was changed, in some cases to increase C. difficile growth (15). Based on these observations from their bottom-up approach, it is unclear how more complex combinations would affect the inhibition of C. difficile. So instead, we sought to find an inhibitory community using a top-down approach and to begin with an inhibitory community. In a recent top-down approach, Auchtung et al. developed a set of reduced communities from human fecal communities that were grown in minibioreactor arrays and tested for inhibition first *in vitro* and then in a mouse model (16). They found four reduced communities that were able to reduce C. difficile colonization but with varied effects in a mouse model with the same gut microbiota. One way they reduced the community was by dilution of the initial fecal sample. In our experiments, we began with a fresh whole fecal community to first determine if inhibition was possible. In the conditions under which C. difficile was inhibited, with cefoperazone and streptomycin, we diluted the FCT to determine the minimal community which maintained inhibition. Cefoperazone-treated mice were unable to maintain inhibition of C. difficile with diluted FCT pretreatments, and C. difficile remained colonized. Streptomycin-treated mice were able to maintain inhibition with diluted FCT pretreatment. While the diluted FCTs had similar diversities and bacterial abundances, the differences in effect on C. difficile revealed the minimal changes associated with either colonization resistance or clearance.

We previously hypothesized that mice treated with either clindamycin, cefoperazone, or streptomycin would not have the same bacterial community changes associated with C. difficile clearance (14). In that set of experiments, the dose of the antibiotic was varied to titrate changes to the community and to determine what changes allow C. difficile to colonize and then be spontaneously cleared. We observed antibiotic-specific changes associated with C. difficile clearance. The data presented here complement those observations. For clindamycin-treated mice, there was no difference in colonization, clearance, or relative abundance between PBS and FCT pretreatment. C. difficile had similar colonization dynamics. It is possible that there was insufficient time for the FCT to engraft. However, when we added more time between clindamycin treatment and C. difficile challenge, C. difficile was unable to colonize (data not shown). Therefore, clindamycin-treated mice appeared to have been naturally recovering inhibition to C. difficile, which was unaffected by the FCT pretreatment. For cefoperazone-treated mice, the FCT pretreatment enabled the gut microbiota to eliminate the colonization but only at its most concentrated dose. This observation supports our previous discussion (14), indicating that the cefoperazone-treated community is more sensitive to the amount of FCT it receives, since cefoperazone reduced many bacterial groups and associations (Fig. S6). As we previously hypothesized, streptomycin-treated mice were enabled to clear C. difficile with a subset of the community, with the FCT pretreatment diluted 1:10^3^. Since we titrated the FCT dilutions, we could compare the bacterial communities of the mice that gained the ability to clear C. difficile to the mice receiving the next dilution which could not to elucidate the minimal relative abundance differences. In agreement with previous studies, OTUs related to *Lachnospiraceae*, *Porphyromonadaceae*, and *Ruminococcaceae* increased with the clearance of C. difficile in the streptomycin-treated mice (14, 17–21). These data agree with our previous hypothesis that a reduced fecal community would be able to promote clearance of C. difficile only in streptomycin-treated mice.

10.1128/mbio.01364-22.6 FIG S6 Murine gut bacterial community OTUs of cefoperazone-treated mice at the time of challenge. Murine gut bacterial community OTUs that were present in at least one sample at the time of C. difficile challenge (day 0) are shown. Mice were pretreated with either fecal community transplant (FCT; open circles) or FCT diluted 1:10 (filled circles). Points represent individual samples. A dashed vertical line indicates the limit of detection. OTUs are ordered alphabetically. An asterisk indicates that the OTU was unclassified at a lower classification rank. Download FIG S6, TIF file, 0.5 MB. Copyright © 2022 Lesniak et al. 2022 Lesniak et al. https://creativecommons.org/licenses/by/4.0/ This content is distributed under the terms of the Creative Commons Attribution 4.0 International license.

In addition to clearing C. difficile, a reduced fecal community restored colonization resistance to streptomycin-treated mice. Mice that received FCT pretreatment as dilute as 1:10^2^ were not colonized to a detectable level. While restoring colonization resistance is not novel (22), here we have shown that the restoration of colonization resistance is dependent on the community perturbation and the fecal community being transplanted. As we identified community members associated with clearance, OTUs related to *Akkermansia*, *Olsenella*, and *Porphyromonadaceae* were more abundant and an OTU related to *Enterobacteriaceae* was less abundant at the time of C. difficile challenge. *Enterobacteriaceae* has been associated with C. difficile colonization and inflammation (14, 23, 24). Larger populations of *Akkermansia* were associated with preventing colonization, which we had previously observed, potentially indicating the maintenance of a protective mucus layer (14, 25–27). Increased populations of a select set of OTUs related to *Porphyromonadaceae* were also more abundant in mice that were resistant to colonization. *Porphyromonadaceae* may inhibit C. difficile via butyrate and acetate production, which has been associated with successful FMT treatments (28–30). Different populations of OTUs associated with *Porphyromonadaceae* were associated with colonization resistance and with colonization clearance. These colonization resistance-associated OTUs (OTUs 8, 25, and 29) may have OTU-specific functions or OTU-dependent abundances of members of the community, such as *Akkermansia* and *Enterobacteriaceae*. With our top-down approach, we reduced the number of gut bacterial community members that were associated with colonization resistance in streptomycin-treated mice.

Further investigation into the heterogeneity of CDIs will help to elucidate the niche range of C. difficile and the interventions to eliminate them. Here, we were limited by our experimental design and methods to refining our understanding of colonization resistance restoration in streptomycin-treated mice. Future studies can expand beyond the presence and abundance of the bacterial groups and investigate the metabolites and host immune response. A refined understanding of the bacteria, metabolites, and host response can help develop more-targeted therapies to restore C. difficile colonization resistance. Additionally, building up the experimental design to incorporate more FCT treatment variations or inoculation regimens could expand our understanding of the necessary components for colonization resistance for each antibiotic treatment. We designed our experiments to closely match previous mouse models for CDI and added days prior to C. difficile challenge for the FCT treatment (14, 31, 32). It may be possible to restore colonization resistance to clindamycin or cefoperazone if the antibiotic treatment, recovery period, and FCT treatment were modified to allow the FCT to have an effect. Other methods could be used to make the mice susceptible to CDI and then tested for the effectiveness of the FCT treatment (18, 33). Further modification and characterization of the fecal communities could reduce the necessary community members and metabolites to promote colonization resistance. The results from these additional studies could expand upon our limitations and reveal specific bacterial communities that could restore C. difficile colonization resistance for each susceptibility.

We have demonstrated that a reduced bacterial community can restore colonization resistance, but the effect of the community and the bacteria that colonized were dependent on the specific changes to the community that were caused by each antibiotic. When the fecal community was transplanted into antibiotic-induced susceptible mice, only mice treated with streptomycin were able to restore colonization resistance. Previous studies have identified reduced communities in a murine model using a homogeneous gut microbiota with a bottom-up approach (7, 19). Treatments supplementing the gut microbiota would benefit from being tested in different communities susceptible to CDI. Further research is necessary to characterize the specific niche spaces of C. difficile-susceptible communities and the specific requirements to fill those spaces. It may then be possible to identify people with gut microbiota that are susceptible to CDI and develop targeted reduced bacterial communities to recover colonization resistance and reduce the risk of CDI.

## MATERIALS AND METHODS

### Animal care.

Mice used in experiments were 6- to 13-week-old conventionally reared SPF male C57BL/6 mice obtained from a single breeding colony at the University of Michigan. During the experiment, mice were housed with two or three mice per cage. All murine experiments were approved by the University of Michigan Animal Care and Use Committee (IACUC) under protocol number PRO00006983.

### Antibiotic administration.

Antibiotics were chosen and administered based on previous studies (14, 31, 32). Cefoperazone, clindamycin, and streptomycin treatment produced diverse communities and responses to CDI. Mice were given either cefoperazone, clindamycin, or streptomycin. Cefoperazone (0.5 mg/mL) and streptomycin (5 mg/mL) were administered via drinking water *ad libitum* for 5 days, beginning 9 days prior to C. difficile challenge. Antibiotic water was replaced every 2 days. Clindamycin (10 mg/kg) was injected into the intraperitoneal space 2 days prior to challenge with C. difficile. All antibiotics were filter sterilized with a 0.22-μm syringe filter prior to use.

### FCTs.

Fecal pellets were collected from similarly aged C57BL/6 mice not being used in an experiment the day of the fecal community transplants. Fifteen to 20 pellets were collected and weighed. The fecal pellets were homogenized weight per weight in phosphate-saline buffer (PBS) containing 15% glycerol (fecal community transplant [FCT]) under anaerobic conditions. The FCT was serially diluted in PBS containing 15% glycerol down to a 1:10^5^ fecal dilution and aliquoted into tubes for administration by gavage into mice. One set of aliquots was frozen at −80°C to be used the following day for the cefoperazone and streptomycin experiments. Frozen aliquots were thawed at 30°C for 5 min prior to being used. All fecal community dilutions were centrifuged at 7,500 rpm for 60 s, and the supernatant was used for inoculation. Mice were inoculated with 100 μL of the fecal dilution orally via a 21-gauge gavage needle. Fecal community transplants were administered from the most dilute to the least, which began with mice receiving PBS and finished with mice receiving FCT. Aliquots were frozen at −80∘C after use for sequencing. These experiments were repeated 8 times with a different starting source each time. This method was adapted from our previous study (18).

### 16S rRNA quantitative real-time PCR.

Quantitative analysis of 16S rRNA in fecal community dilutions used for FCT was carried out using quantitative real-time PCR with primers and cycler conditions specified previously (34). Reaction volumes were prepared using 6 μL of SYBR Green PCR master mix (Applied Biosciences; reference no. 4344463), 1 μL each of forward and reverse primers, and 2 μL of sample DNA template. All qPCRs were run on a LightCycler 96 (Roche; reference no. 05815916001) using instrument-specific plates and seals.

### C. difficile challenge.

For experiments using streptomycin or cefoperazone, mice were given untreated drinking water for 96 h before challenge with C. difficile strain 630Δerm spores. For experiments using clindamycin, mice were given untreated drinking water for 48 h, from the time of the intraperitoneal injection until the challenge with C. difficile strain 630Δerm spores. This time frame was designed to closely replicate the previous mouse model (14, 31, 32), with the insertion of a day (clindamycin) or two (cefoperazone and streptomycin) before inoculation of the mice with the fecal communities. C. difficile spores were aliquoted from a single spore stock stored at 4°C. Spore concentration was determined 2 days prior to the day of challenge (35). Mice were inoculated with 10^3^ C. difficile spores via oral gavage. After inoculation of the mice, the remaining spore solution was serially diluted and plated to confirm the spore concentration.

### Sample collection.

Fecal samples were collected prior to antibiotic administration, after the antibiotics were removed, prior to C. difficile challenge, and on each of the 10 days postinfection. Approximately 15 mg of each fecal sample was collected and weighed for plating C. difficile CFU, and the remaining sample was frozen at −80°C for later sequencing. The weighed fecal samples were anaerobically serially diluted in PBS, plated on taurocholate cycloserine cefoxitin fructose agar (TCCFA) plates, and incubated at 37°C for 24 h. The resultant colonies were enumerated to determine the C. difficile CFU (36).

### DNA sequencing.

Total bacterial DNA was extracted from the frozen samples by the MOBIO PowerSoil-htp 96-well soil DNA isolation kit. We amplified the 16S rRNA gene V4 region, and the amplicons were sequenced on an Illumina MiSeq system as described previously (37).

### Sequence curation.

Sequences were processed using mothur (v.1.44.1) (37, 38). We used a 3% dissimilarity cutoff to group sequences into operational taxonomic units (OTUs) and a naive Bayesian classifier with the Ribosomal Database Project training set (version 16) to assign taxonomic classifications to OTUs (39). We sequenced a mock community of known 16S rRNA gene sequences and composition. We processed this mock community in parallel with our samples to determine the error rate for our sequence curation, which was 0.029%.

### Statistical analysis and modeling.

We calculated diversity metrics in mothur. For α-diversity comparisons, we calculated the number of OTUs (*S*_obs_) and the inverse Simpson diversity index. For β-diversity comparisons, we calculated dissimilarity matrices based on the metric of Yue and Clayton (θ_YC_) (40). We averaged 1,000 subsamples of 2,480 counts/sample, or rarefied them, to limit uneven sampling biases. We tested for differences in relative abundance between outcomes with LEfSe in mothur (41). All other statistical analyses and data visualization were completed in R (v4.0.5) with the tidyverse package (v1.3.1). Pairwise comparisons of α-diversity (*S*_obs_ and inverse Simpson diversity index) and β-diversity (θ_YC_) were calculated by a pairwise Wilcoxon rank sum test. Correlations between bacterial genera and fecal community dilution were calculated using the Spearman correlation. *P* values were corrected for multiple comparisons with a Benjamini and Hochberg adjustment for a type I error rate of 0.05 (42). For streptomycin experiments, conditional independence networks were calculated from the day 1 through day 5 samples of all mice using SPIEC-EASI (sparse inverse covariance estimation for ecological association inference) methods from the SpiecEasi R package after optimizing lambda to 0.001 with a network stability between 0.045 and 0.05 (v1.0.7) (43).

### Data availability.

Scripts necessary to reproduce our analysis and this paper are available in an online repository (https://github.com/SchlossLab/Lesniak_restoreCR_mBio_2022). All 16S rRNA gene sequence data and associated metadata are available through the Sequence Read Archive via accession no. SRP373949.
