# Supplementary figures and images for: Diluted Fecal Community Transplant Restores Clostridioides difficile Colonization Resistance to Antibiotic-Perturbed Murine Communities
Source: mBio. 2022 Aug 1;13(4):e01364-22. doi: 10.1128/mbio.01364-22 (PMC9426422; doi:10.1128/mbio.01364-22)

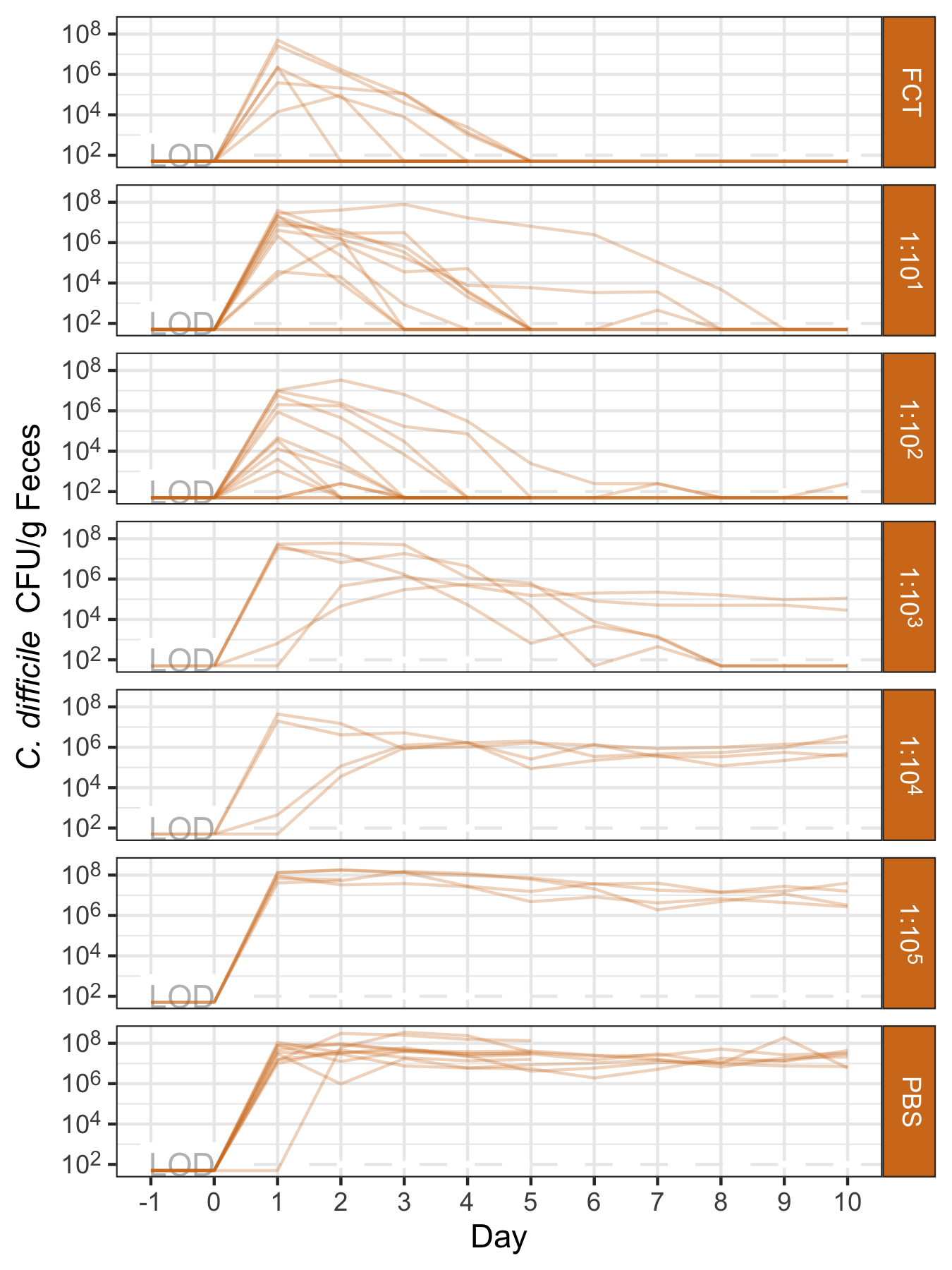

Supplement: FIG S1 [file mbio.01364-22-s0001.tif]

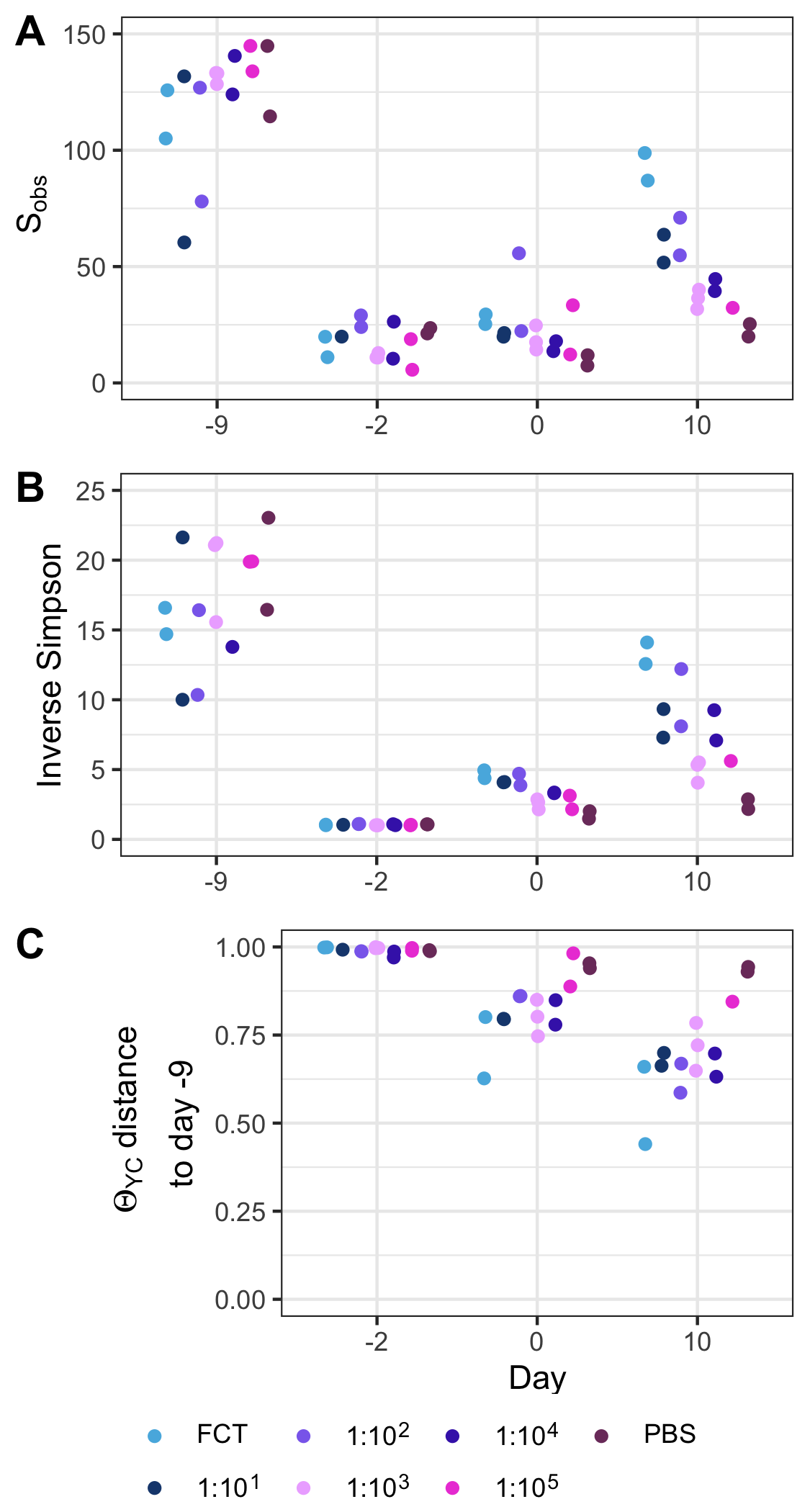

Supplement: FIG S3 [file mbio.01364-22-s0003.tif]

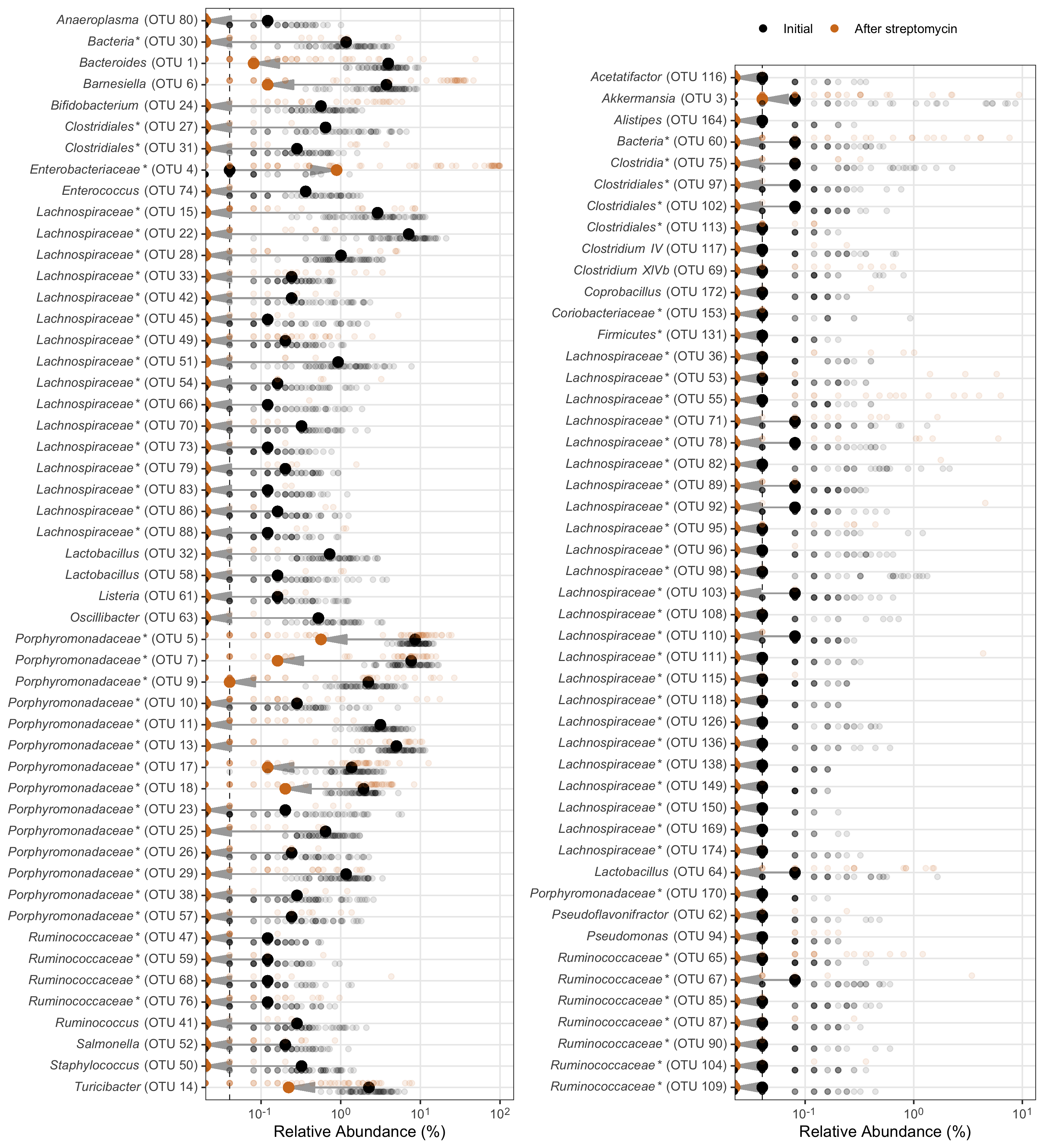

Supplement: FIG S5 [file mbio.01364-22-s0005.tif]

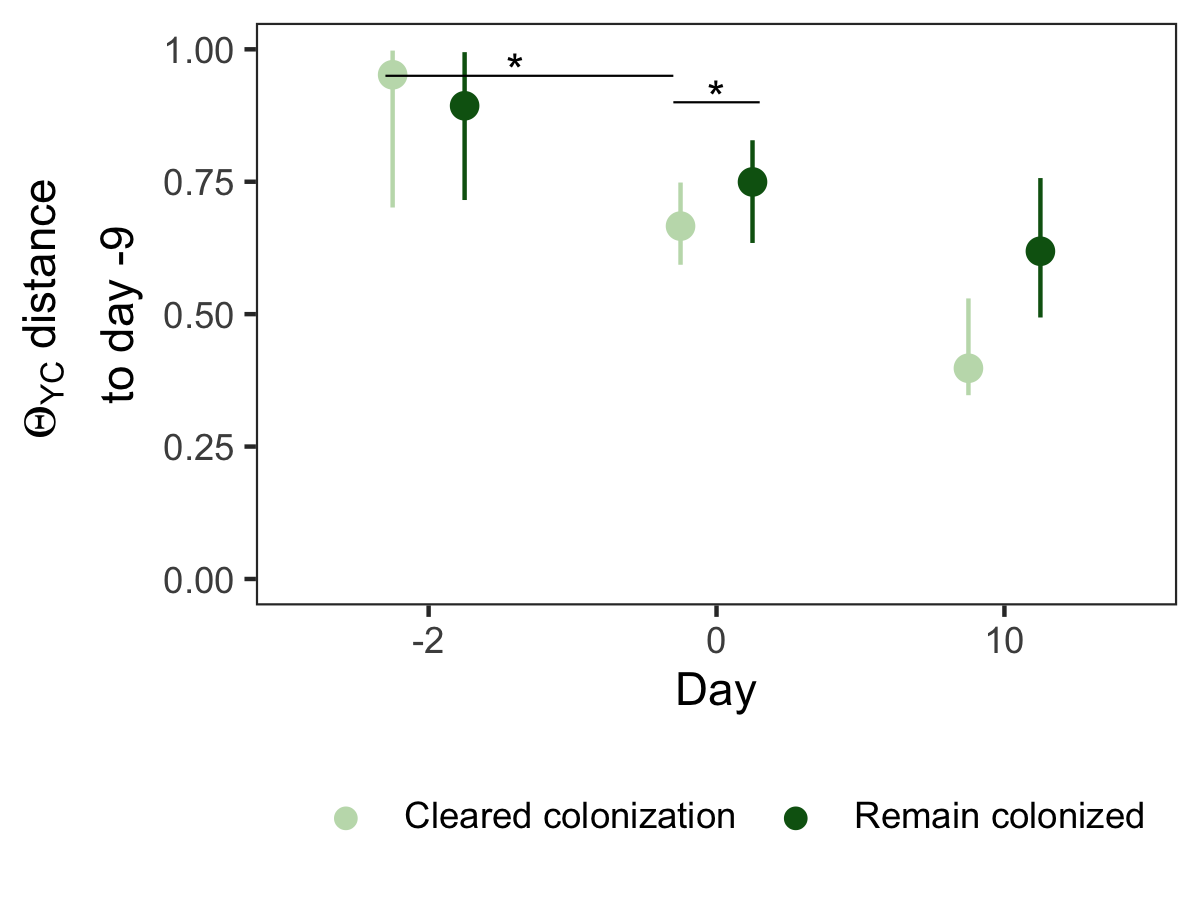

Supplement: FIG S4 [file mbio.01364-22-s0004.tif]

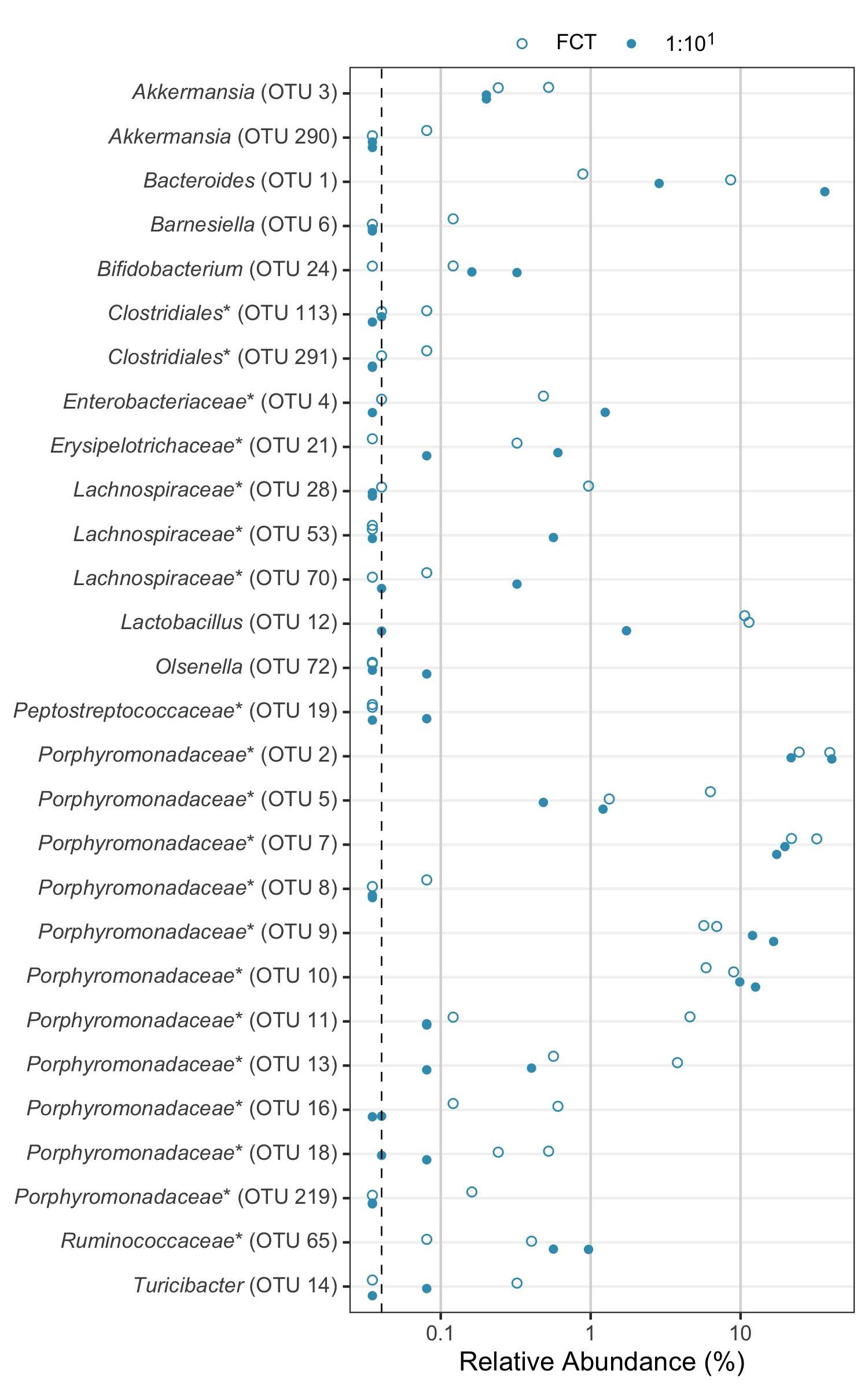

Supplement: FIG S6 [file mbio.01364-22-s0006.tif]
